# Does the advice requested by carers of people who live with dementia reflect the level of commissioned post-diagnostic support? A retrospective evaluation of calls to the Me2U dementia day centre 24-hour advice line

**DOI:** 10.1192/bjo.2021.884

**Published:** 2021-06-18

**Authors:** Rajan Nathan, Stephen Callaghan, Kelly Walker, Angela Mason, Rosemarie Whittington

**Affiliations:** 1 Cheshire and Wirral Partnership NHS Foundation Trust; 2 EQE Health Ltd; 3 Me2u Dementia Day Centre

## Abstract

**Aims:**

The aim was to examine the reasons for advice requests by carers of people who live with dementia (PLWD) that attend the Me2u dementia day centre in order to identify key explanatory themes. We hypothesised that requests were related mainly to coordinating care and clinical issues due to limited post-diagnostic support (PDS) in our area.

**Background:**

The Me2u dementia day centre (Merseyside) cares for PLWD and also supports carers. As part of the service, a 24-hour advice line is included for PLWD and their carers who attend the centre. Locally, there is limited PDS and most carers navigate the health and social care system alone mirroring the findings by the National Collaborating Centre for Mental Health (NCCMH).

**Method:**

We undertook a retrospective evaluation of 244 advice calls, from 64 carers, between 01/06/2019 and 31/12/2019. We analysed time of call, type of advice, type of dementia, age and whether the advice was for the PLWD or for the carer.

**Result:**

Of the 244 calls, the most common time to call was between 09.00 - 14.00 (n = 168; (68.8%) peak 09.00 - 10.00 (n = 38). Average age of the person about whom the advice was sought was 79.08 years. 91.4% of the advice calls related to PLWD (most common dementia Alzheimer's) and 8.6% to the carer only. The mean number of calls per person was 3.8 (range 1–24).

Advice data were grouped into 9 broad themes namely, related to symptoms/behaviour (32.79%, n = 80), request for Me2u to coordinate care (20.08%, n = 49), general advice (14.75%, n = 36), personal care (9.42%, n = 23), carer only advice (8.60%, n = 21), social issues (6.14%, n = 15), social care (4.50%, n = 11), safeguarding (2.46%, n = 6), non-health and social care issue (1.23%, n = 3).

**Conclusion:**

Reasons for limited/poor PDS given by the NCCMH are; absence of named coordinators of care, over-reliance on families and carers to manage and facilitate appointments, poor recognition and management of comorbidities. This data show that 52.87% of calls were for clinical advice and coordination of care reflecting NCCMH findings. The interventions post-call reduced the impact on providers of urgent care.

These findings provide support for the provision of a [24-hour] advice line as a routine part of post-diagnostic support services, especially in areas that have limited or poor PDS. Commissioners of PDS services in areas that have limited or poor PDS should make this a priority to prevent unplanned admissions to hospital and carer breakdown.

